# Comparison of Six Handheld Ultrasound Devices by Pediatric Point of Care Ultrasound (POCUS) Experts

**DOI:** 10.24908/pocusj.v10i01.18722

**Published:** 2025-04-15

**Authors:** Stephanie K. Leung, Ria Dancel, Riya N. Soni, Ariadna Perez-Sanchez, Michael J. Mader, Haitham Al-Wahab, Thomas W. Conlon, Maria V. Fraga, Javier J. Lasa, Andrea Matho, Hannah Smith, Nilam J. Soni

**Affiliations:** 1 Department of Pediatrics, Division of Emergency Medicine, Texas Children's Hospital, Baylor College of Medicine, Houston, Texas, USA; 2 Department of Pediatrics, Division of Pediatric Hospital Medicine, University of North Carolina, Chapel Hill, North Carolina, USA.; 3 Department of Medicine, Division of Hospital Medicine, University of North Carolina, Chapel Hill, North Carolina, USA.; 4 Section of Pulmonary Medicine, South Texas Veterans Health Care System, San Antonio, Texas, USA; 5 Division of Hospital Medicine, Joe R. Teresa Lozano Long School of Medicine, University of Texas Health San Antonio, San Antonio, Texas, USA; 6 Research and Development Service, South Texas Veterans Health Care System, San Antonio, Texas, USA; 7 Department of Pediatrics, UTHealth McGovern Medical School, Houston, Texas, USA; 8 Department of Anesthesiology and Critical Care Medicine, The Children's Hospital of Philadelphia, Perelman School of Medicine, University of Pennsylvania, Philadelphia, Pennsylvania, USA.; 9 Division of Neonatology, Department of Pediatrics, Children's Hospital of Philadelphia, Perelman School of Medicine, University of Pennsylvania, Philadelphia, Pennsylvania, USA.; 10 Division of Cardiology and Critical Care, Department of Pediatrics, UT Southwestern Medical Center, Dallas Texas, USA; 11 Division of Hospital Medicine, Department of Pediatrics, Keck School of Medicine of USC; 12 Department of Emergency Medicine, UTHealth McGovern Medical School, Houston, Texas, USA.; 13 Section of Hospital Medicine, South Texas Veterans Health Care System, San Antonio, Texas, USA; 14 Division of Pulmonary Diseases and Critical Care Medicine, Joe R. and Teresa Lozano Long School of Medicine, University of Texas Health San Antonio, San Antonio, Texas, USA

**Keywords:** point of care ultrasound, POCUS, handheld ultrasound, device, pediatrics, emergency medicine

## Abstract

**Background:**

Point of care ultrasound (POCUS) use is increasing among pediatric clinicians, but lack of access to ultrasound devices is a major barrier. The availability of pocket-sized handheld ultrasound devices (“handhelds”) has improved access. However, no head-to-head comparative studies of handhelds in children have been performed to guide purchasing decisions.

**Methods:**

This cross-sectional study compared six handhelds: Butterfly iQ+™ (Butterfly Network Inc.), Clarius® (Clarius Mobile Health™), Kosmos™(EchoNous), TE Air™ (Mindray®), Vscan Air™ SL (General Electric), and Lumify™ (Philips Healthcare). Eight pediatric POCUS experts acquired views showing the abdominal right upper quadrant (RUQ), cardiac apical 4-chamber, and superficial neck and lung on a standardized pediatric model using each handheld. Experts rated each handheld by its image quality, ease of use, and their overall satisfaction.

**Results:**

Vscan Air™, Kosmos™, and Lumify™ were rated highest for ease of use, image quality, and overall satisfaction. Most experts recommended Vscan Air™ for purchase. The five most desirable characteristics of handhelds were image quality, ease of use, total costs, transducer size, and availability of different transducer types. All six handhelds had important advantages and disadvantages per experts, and no single device had all the desired characteristics.

**Conclusions:**

Pediatric POCUS experts rated Vscan Air™, Kosmos™, and Lumify™ highest for ease of use, image quality, and overall satisfaction. No device was clearly superior across all applications. Subjective characteristics, particularly probe ergonomics, may be a deciding factor when selecting handhelds in pediatrics. There is a need to develop handhelds specifically for use in pediatric populations.

## Background

Point of care ultrasound (POCUS) is an essential tool for pediatric clinicians. Handheld ultrasound devices (handhelds) have been developed with enhanced portability to increase access and affordability. Handheld usage in pediatrics was first described in a neonatal intensive care unit in 2002 [1]. Since then, several pediatric studies have described handheld utilization, mainly for rheumatic heart disease and cardiac function assessment in resource-limited settings. Often, non-physician healthcare providers or parents were the operators [2–10]. Other established pediatric applications of handhelds include bladder [11], muscle [12], thyroid gland [13], vasculature [14], and vocal cord evaluation [15].

Despite the established utility of handhelds for performing POCUS exams in children, relatively few pediatric clinicians have access to ultrasound devices. Lack of access to ultrasound devices continues to be a top barrier to POCUS use in pediatrics [16, 17]. Even though cart-based ultrasound machines generally have superior image quality and additional features, the availability of affordable pocket-sized handhelds has improved pediatric clinicians' access to ultrasound. However, few studies have compared the performance of different handhelds in children. Past pediatric studies compared handhelds vs. cart-based ultrasound machines, rather than comparing handhelds against one another [2, 3, 5–7, 9, 14, 18]. Even in adults, few studies with head-to-head comparisons of handhelds have been conducted [19–22]. Currently, no head-to-head comparison of different handhelds in pediatrics has been conducted [3, 14, 18–20].

The primary objective of this study was to compare the image quality, ease of use, and overall satisfaction of six handhelds available in the United States when used by pediatric POCUS experts. Additionally, we sought to identify the most and least important characteristics of common handhelds per pediatric POCUS experts. We hypothesized that current handhelds developed for adult populations may provide adequate imaging, but transducer size and image resolution may not be optimized for children. Our findings can guide pediatric clinicians and institutions to select and purchase handhelds for pediatric use.

## Methods

### Study Design, Setting, and Participants

We conducted a cross-sectional study during a two-day POCUS continuing medical education course in January 2024. While this study focused on comparing handhelds for use in pediatric specialties, our group previously published a similar comparative study in adult specialties [22]. It utilized the same protocol and processes for data collection and analysis that are described below [22].

Eight pediatric POCUS experts—physicians specializing in pediatric cardiology, critical care, emergency medicine, hospital medicine, and neonatology—participated. These pediatric POCUS experts were selected based on their past training in pediatric ultrasound, through a dedicated POCUS fellowship or having completed a POCUS certificate program, and experience teaching pediatric POCUS at national courses. The University of Texas Health San Antonio Institutional Review Board deemed this study as non-regulated research involving human subjects (STUDY00000326).

### Protocol

Six handhelds with low- and high-frequency transducers that are commercially available in the United States were compared: Butterfly iQ+™ (Butterfly Network, Inc.) all-in-one transducer (referred to as “Butterfly iQ+™”) connected by a Lightning® cable to an Apple iPad® (iPad Pro®, iPad Air®); Clarius® (Clarius Mobile Health™) L15 and PA transducers (referred to as “Clarius®”) connected wirelessly to an Apple iPad®; Kosmos™ (EchoNous, Inc.) linear-array (Lexsa) and phased-array (Torso-one) transducers (referred to as “Kosmos™”) connected by a USB-C cable to an Apple iPad®; TE Air™ (Mindray®) phased-array transducer (referred to as “Mindray®”) connected wirelessly to an Apple iPad® and Apple iPhone® (iPhone 11 Pro®); Lumify™ (Philips Healthcare) linear array and phased array transducers (referred to as “Lumify™”) connected by a USB-C cable to a Samsung Galaxy tablet™; and Vscan Air™ SL (GE Healthcare) sector phased-array and linear-array transducer (referred to as “Vscan Air™”) connected wirelessly to a Samsung Galaxy S7 tablet ™. Eight companies were invited to participate by providing handhelds. The eight handheld companies were selected based on consensus of the adult (n=35) and pediatric (n=8) POCUS experts, who were asked which handhelds are most used in the United States. Exo, Vave Health, and Butterfly Network, Inc. declined to participate. Butterfly IQ+™ equipment was loaned by three study investigators, but Exo and Vave handhelds were not available for inclusion.

Pediatric POCUS experts scanned a 6-year-old female standardized patient with average height and weight using all six handhelds. Three standard POCUS views were acquired, including 1) Focused Assessment with Sonography in Trauma (FAST) right upper quadrant (RUQ) view (diaphragm, liver, right kidney, and hepatorenal recess), 2) apical 4-chamber view of the heart, and 3) superficial view of the right neck (thyroid, internal jugular vein, and common carotid artery) and lung along the anterior chest wall (ribs, pleural line with lung sliding). The standardized patient was pre-scanned using a cart-based ultrasound machine (Sonosite PX ™ Fujifilm-Sonosite) to ensure high-quality images could be easily obtained.

The eight pediatric POCUS experts independently acquired the same views using all six handhelds on the same standardized patient. A phased-array transducer was used for the RUQ view, except for Butterfly™, which had an all-in-one transducer. All RUQ views were acquired with an abdominal preset and focused on the liver, kidney, diaphragm, aorta, and spine. Color flow Doppler was applied over the vessels in the renal pelvis. For the apical 4-chamber view, a phased-array transducer with a cardiac preset was used. It acquired views of the mitral valve, aortic valve, and right and left atria and ventricles, focusing on the resolution of the endocardial lining and cardiac motion. Color flow Doppler was then applied over the mitral valve and left ventricular outflow tract. For the transverse view of the neck and superficial view of the lung, a high-frequency linear transducer with a venous or vascular preset was used to acquire transverse views of the internal jugular vein, common carotid artery, and thyroid gland. Color flow Doppler was applied over the common carotid artery and internal jugular vein. Next, a lung preset was used to acquire longitudinal views of the lung on the anterior chest wall to visualize lung sliding. All handhelds, except Mindray®, had a high-frequency linear transducer or lung preset.

### Data Collection

This study was conducted in two phases. In real-time, experts first rated the image quality of the three views using each handheld on a scale of 0 (“poor”) to 3 (“excellent”). Specific characteristics were provided to rate the image quality of each view (Appendices 1-3). An overall ranking of each device from 1 (“best”) to 6 (“worst”) was performed. Second, data were collected on overall ease of use, image quality, and satisfaction of each device (“overall survey”) (Appendix 4). For ease of use, experts rated physical characteristics, software navigation, maneuverability of the transducer and tablet for imaging, and overall satisfaction. For image quality, experts rated detail resolution, contrast resolution, penetration, clutter, and overall satisfaction. The overall ranking assessed satisfaction and recommendation for purchase. Ratings utilized standardized statements on a Likert scale of 1 (“strongly disagree” or “very dissatisfied”) to 5 (“strongly agree” or “very satisfied”). Qualitative feedback was collected using free text. Experts completed all data collection forms immediately, and no later than 72 hours, after scanning the standardized patient. All data were captured electronically using REDCap™ (Vanderbilt University, Nashville, TN, USA).

### Data Analysis

Descriptive statistics of the POCUS experts were reported as frequencies with percentages, without any statistical analysis. Ratings of ease of use and image quality were compared using the Kruskal-Wallis rank sum test, with the Dwass-Steel-Critchlow-Fligner post hoc method to control the family-wise error rate. Rank analysis was performed via Friedman's test, followed by a post-hoc Sign test for paired data, using Holm's step-down procedure to control the family-wise error rate. For image quality ratings, scores were calculated by finding the mean score of each characteristic across raters and then adding the five means within a view. The comparison of devices used a non-linear mixed model to predict rating scores, including the device and view characteristics as fixed factors and rater as a random factor.

Potential bias due to prior experience with handhelds was assessed. Experts rated their experience with a device as none (1), some (2), or proficient (3). Spearman correlation coefficients were calculated to evaluate the correlation between experts' prior experience with each handheld and their ratings for ease of use, image quality, and overall satisfaction. A modified independent sample t-test was used to test for statistical significance. A p-value <0.05 denoted statistical significance. All analyses were performed with SAS software version 9.4.

Free text responses were analyzed using a qualitative deductive and inductive coding process based on a framework method approach. Advantages and disadvantages of handhelds were coded and tabulated using a framework for free text responses. Questions about coding were resolved by discussion between two investigators for final code assignment.

## Results

### POCUS Experts

Eight pediatric POCUS experts who were physicians specializing in pediatric cardiology, critical care, emergency medicine, hospital medicine, and neonatology acquired and rated RUQ, apical 4-chamber, and superficial neck and lung views using six handhelds. Most POCUS experts were female, with greater than five years of clinical practice and POCUS experience. All experts had experience teaching POCUS at national conferences (Table 1).

**Table 1.** Characteristics of Pediatric Point of Care Ultrasound (POCUS) Experts

Characteristic	All Experts (%) n=8
**Gender**	
Female	5 (63)
Male	3 (37)
**Pediatric Specialty**	
Cardiology & Critical Care	3 (38)
Emergency Medicine	2 (25)
Hospital Medicine	2 (25)
Neonatology	1 (12)
**Experience in Clinical Practice**	
0-5 years	1 (12)
6-10 years	5 (63)
>10 years	2 (25)
**Experience Using POCUS**	
0-5 years	1 (12)
6-10 years	4 (50)
>10 years	3 (38)
**POCUS Applications Routinely Used^1^**	
Cardiac	8 (100)
Pulmonary	8 (100)
Abdomen	7 (88)
Procedural Guidance	7 (88)
Vascular	6 (75)
Skin/soft tissues	4 (50)

1 Experts could select >1 application and each application represents a percentage of eight experts.

POCUS, point of care ultrasound.

### Handheld Characteristics

Characteristics of the six handhelds compared in this study are shown in Table 2. All handhelds had M-mode, color flow Doppler, and pulsed-wave Doppler imaging modes, but only Kosmos™ had continuous-wave Doppler. All handhelds, except Mindray®, were compatible with both iOS and Android tablets. Clarius™, Mindray®, and Vscan Air™ were wireless. Two were all-in-one devices (Butterfly iQ+™ had one transducer with multiple exam presets; Vscan Air™ was a dual probe with opposing phased- and linear-array transducers).

**Table 2.** Characteristics of Handheld Ultrasound Devices

	MODES^1^						PROBE TYPES & CHARACTERISTICS						STUDY VIEWS			APPROXIMATE COST (per probe)^2^
	MM	CFD	PD	TDI	PWD	CWD	All-in-one	Size	Weight	Wired	Wireless	iOS vs. Android	Abdomen RUQ view	Cardiac A4C view	Superficial Neck/Lung view	
**Butterfly**																
Butterfly iQ+	✓	✓	✓		✓		✓	56 × 35 × 163 mm	309 g	✓		iOS + Android	✓	✓	✓	$3,500 or $2,700 + $420/yr
**Clarius**																
Phased Array (PA HD3)	✓	✓	✓		✓			148 × 76 × 32 mm	292 g		✓	iOS + Android		✓		$3,600 - $5,400 + $595/yr
Linear (L15 HD3)	✓	✓	✓		✓			147 × 76 × 32 mm	290 g		✓				✓	
Convex (C3 HD3)	✓	✓	✓		✓			146 × 76 × 32 mm	308 g		✓		✓			
**Kosmos**																
Linear (Lexsa)	✓	✓	✓		✓			155 × 56 × 35 mm	280 g	✓		iOS + Android			✓	$4,500
Phased-array (Torso-one)	✓	✓	✓	✓	✓	✓		150 × 56 × 35 mm	275 g	✓			✓	✓		
**Lumify**																
Sector (S4-1)	✓	✓			✓			102 × 55 mm	96 g	✓		iOS + Android		✓		$5,250
Linear (L12-4)	✓	✓			✓			114 × 45 mm	108g	✓					✓	
Curved (C5-2)	✓	✓			✓			114 × 45 mm	136 g	✓			✓			
**Mindray**																
Mindray TE Air	✓	✓	✓	✓	✓		✓	33 × 47 × 170 mm	198 g		✓	iOS	✓	✓		$6,000 - $8,000
**Vscan Air**																
Sector-phased array + Linear (SL)	✓	✓			✓			141 × 67 × 33 mm	218 g		✓	iOS + Android		✓	✓	$4,500
Curved + Linear (CL)	✓	✓			✓			131 × 64 × 31 mm	205 g		✓		✓		✓	

1 Imaging modes in addition to 2-dimensional or B-mode.

2 Approximate cost per probe in January-February 2024, not including the cost of a tablet.

Abbreviations: MM, M-mode; CFD, color-flow Doppler; PD, Power Doppler; TDI, tissue Doppler imaging; PWD, pulsed-wave Doppler; CWD, continuous wave Doppler; RUQ, right upper quadrant; A4C, apical 4-chamber.

### Overall Ease of Use and Image Quality

**Figure 1.**
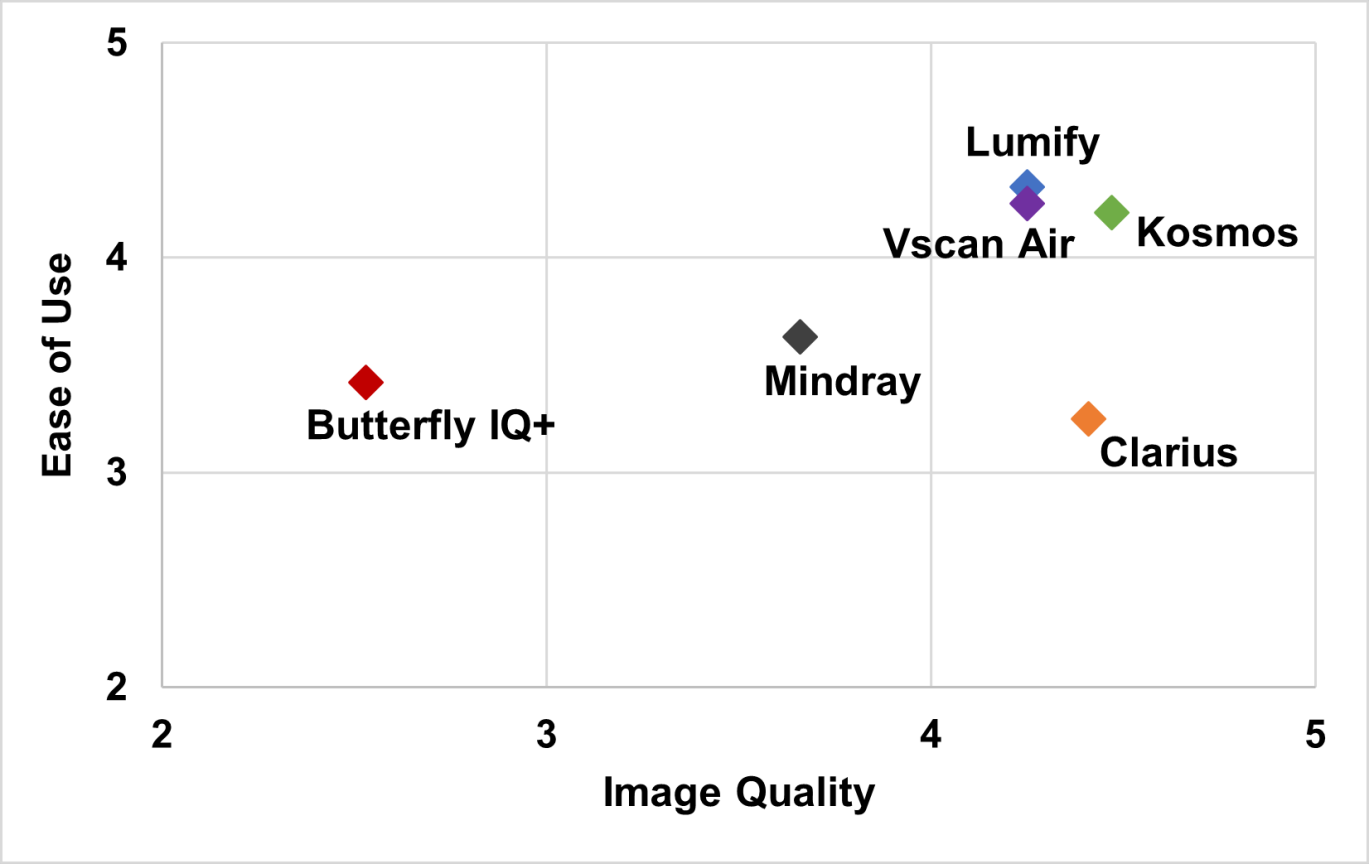
Mean Ratings of Handhelds by Ease of Use and Image Quality

POCUS experts completed an overall survey on handheld image quality, ease of use, and satisfaction. A comparison of mean ratings of ease of use vs. image quality is illustrated in Figure 1. Specific characteristics and ratings for image quality and ease of use are shown in Table 3.

**Table 3.** Overall Ease of Use & Image Quality Ratings of Handheld Ultrasound Devices per Pediatric Expert Users (n=8)

Variable [Mean score (s.d.)]	Butterfly iQ+	Clarius	Kosmos	Lumify	Mindray	Vscan Air	p-value
**Ease of Use**							
**Physical Characteristics**	3.00 (1.7)	2.75 (0.9)	4.13 (1.0)	4.13 (1.4)	4.00 (0.9)	4.00 (1.3)	0.11
**Software**	3.88 (1.2)	3.63 (0.9)	4.38 (0.7)	4.38 (0.7)	3.00 (1.5)	4.38 (0.9)	0.16
**Maneuverability**	3.38 (1.2)	3.38 (1.4)	4.13 (1.0)	4.50 (0.5)	3.88 (1.0)	4.38 (1.1)	0.19
**Overall Satisfaction**	2.88 (1.6)	3.13 (1.2)	4.38 (1.1)	4.38 (1.1)	3.25 (1.2)	4.50 (1.1)	0.031
**Image Quality**							
**Detail Resolution**	2.63 (0.7)	***4.38 (0.7)***	***4.63 (0.7)***	***4.50 (0.5)***	***3.25 (1.2)***	***4.25 (1.0)***	0.0008
**Contrast Resolution**	2.50 (0.5)	***4.38 (0.7)***	***4.38 (0.9)***	***4.38 (0.7)***	***3.38 (1.2)***	***4.25 (1.0)***	0.0016
**Penetration**	2.75 (0.7)	***4.50 (0.5)***	***4.38 (1.1)***	***4.25 (0.7)***	***4.00 (0.9)***	***4.25 (0.7)***	0.0065
**Clutter**	2.25 (0.7)	***4.38 (0.7)***	***4.50 (1.1)***	***3.88 (0.8)***	***4.00 (0.9)***	***4.25 (1.0)***	0.0020
**Overall Satisfaction**	2.00 (1.1)	***4.00 (0.9)***	***4.38 (1.1)***	***4.25 (0.9)***	***3.00 (1.2)***	***4.50 (1.1)***	0.0010

5=Strongly agree; 4=Agree; 3=Neutral; 2=Disagree; 1=Strongly disagree

p-values from Kruskal-Wallis rank sum test; <0.05 indicates at least one device is statistically different from another device.

The highest scoring device in each row and any devices that do not have a significantly different score using the Dwass-Steel-Critchlow-Fligner post hoc method are presented in ***bold italic***.

When comparing overall image quality, Vscan Air™, Lumify™, and Kosmos™ were rated the highest without significant differences between these three handhelds.

Notably, Butterfly iQ+™ scored significantly lower than other devices by >2 points for all image quality parameters (detail resolution, contrast resolution, penetration, clutter, and overall satisfaction).

For overall ease of use, the highest rated devices were Vscan Air™, Lumify™, and Kosmos™. The global test (Kruskal-Wallis) indicated that at least one device was different from the others (p=0.031). However, post hoc pairwise testing with stricter limits for controlling the error rate was unable to distinguish which device had a statistically significant difference in mean score, despite a score difference of 1.62 from the highest to lowest score.

The final survey asked overall satisfaction, ranking of each handheld, and purchasing preference. Vscan Air™, Kosmos™, and Lumify™ received the highest number of “satisfied” responses (Figure 2A), and the same three devices ranked highest from 1 (“best”) to 6 (“worst”) in a slightly different order (Kosmos™, Lumify™, and Vscan Air™) (Figure 2B). Most experts (63%) recommended the Vscan Air™ for purchase (Figure 2C).

**Figure 2A.**
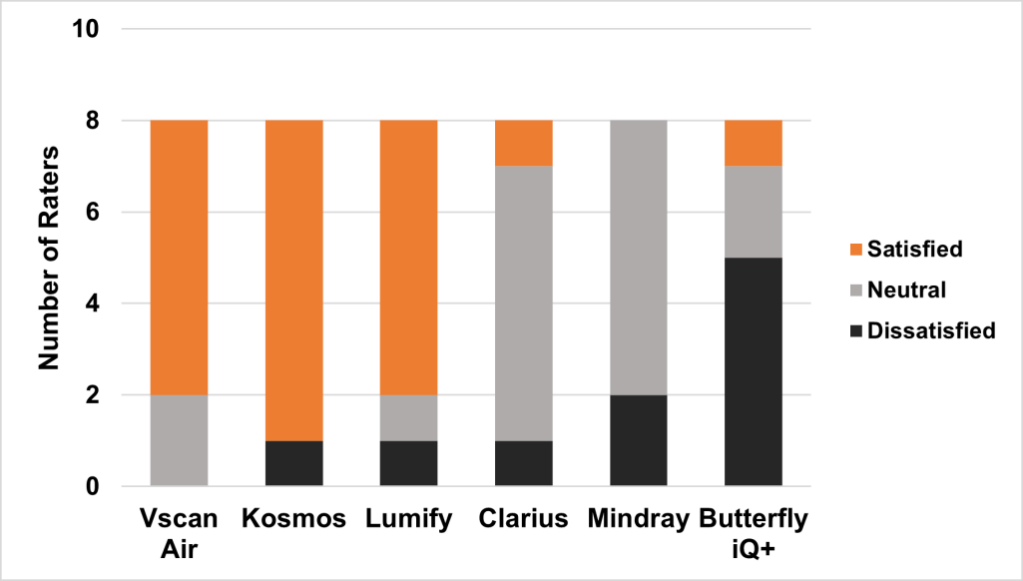
Overall Satisfaction with Each Handheld Device per Pediatric Point of Care Ultrasound Experts

**Figure 2B.**
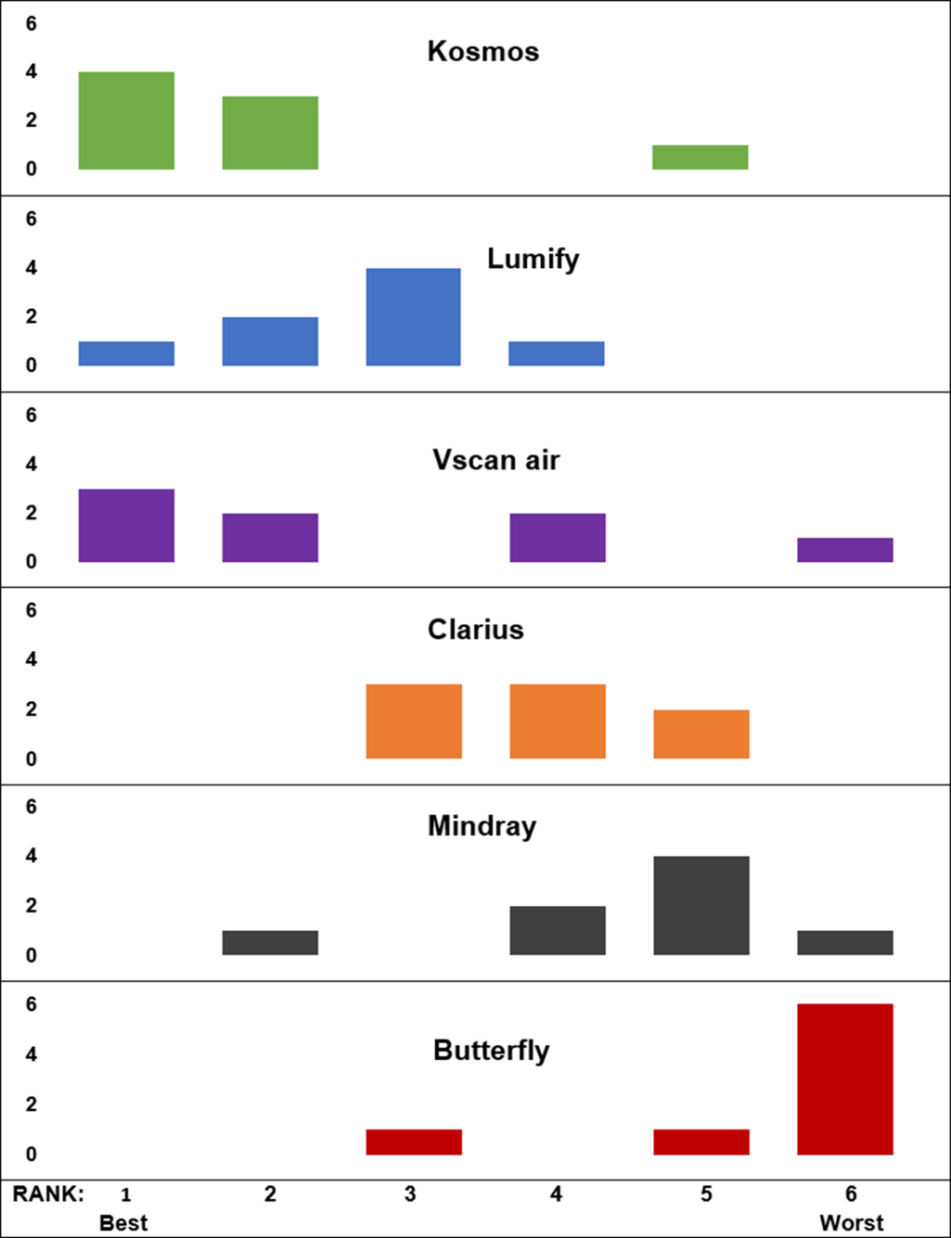
Overall Comparison Rankings of Handhelds by Pediatric Point of Care Ultrasound Experts

**Figure 2C.**
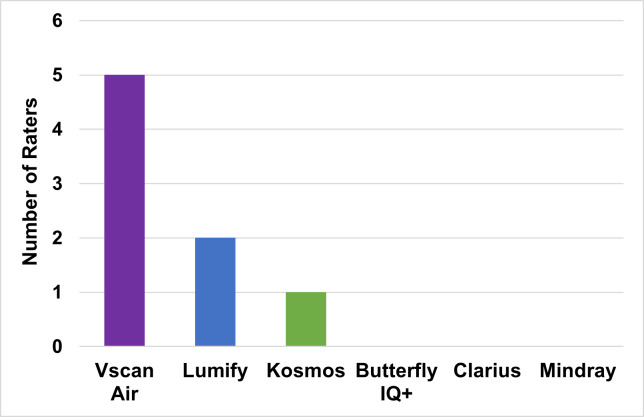
Preferred Handheld to Carry in Pocket by Pediatric Point of Care Ultrasound Experts

Pediatric POCUS experts evaluated the importance of different handheld characteristics (Table 4). The five most important characteristics were image quality, ease of use, total costs, transducer size, and availability of different transducer types. Total cost refers to the cost of the hardware (e.g., handheld transducer, tablet, cables, charger), software, and any associated costs (e.g., subscriptions, warranties). The five least important characteristics were manufacturer's warranty, carrying method (case vs. pocket), reputation of manufacturer, wireless vs. wired connectivity, and inclusion of artificial intelligence technology.

**Table 4.** Importance of Characteristics of Handhelds per Pediatric Point of Care Ultrasound Experts (n=8)

Characteristic	Very Important	Somewhat Important	Not Important
**5 Most Important**			
1. Image Quality	100%	0%	0%
1. Ease of Use	100%	0%	0%
1. Total Costs	100%	0%	0%
2. Transducer Size	88%	12%	0%
2. Availability of Different Transducers	88%	12%	0%
**Intermediate Importance**			
3. Portability	75%	25%	0%
3. Availability of Additional Modes	75%	25%	0%
3. Battery Life	75%	25%	0%
3. Connectivity to any iOS or Android Tablet	75%	25%	0%
4. PACS Integration	75%	12%	12%
5. Option for 1-time purchase	63%	38%	0%
6. Approved by institution	75%	0%	25%
7. Software Calculation Packages	50%	50%	0%
7. Customer Service (prior experience)	50%	50%	0%
**5 Least Important**			
8. Manufacturer's Warranty	38%	63%	0%
9. Carrying Method (case vs. pocket)	25%	63%	12%
10. Reputation of Manufacturer	25%	50%	25%
11. Wireless vs Wired	12%	75%	12%
12. Artificial Intelligence (AI) Technology	12%	38%	50%

PACS, picture archiving and communication system.

### Specific Views

*Abdominal Right Upper Quadrant View.* The RUQ view characteristics that were evaluated included the difference in echogenicity of the renal cortex and liver, clarity of blood vessels in the liver parenchyma, distinction of the medullary pyramids in the renal cortex, far-field resolution, and color flow Doppler of vessels in the renal pelvis (Figure 3; Appendix 5, Supplemental Table 1). For image quality, the handhelds with the highest image quality scores were the Mindray® (12.00), Kosmos™ (11.38), Vscan Air™ (11.38), and Clarius® (11.25) — without a statistically significant difference between the devices. In comparison, Lumify™ and Butterfly iQ+™ had statistically lower total image quality scores. When ranked against each other, Kosmos™, Mindray®, and Vscan Air™ were rated as the top three by most experts.

**Figure 3A.**
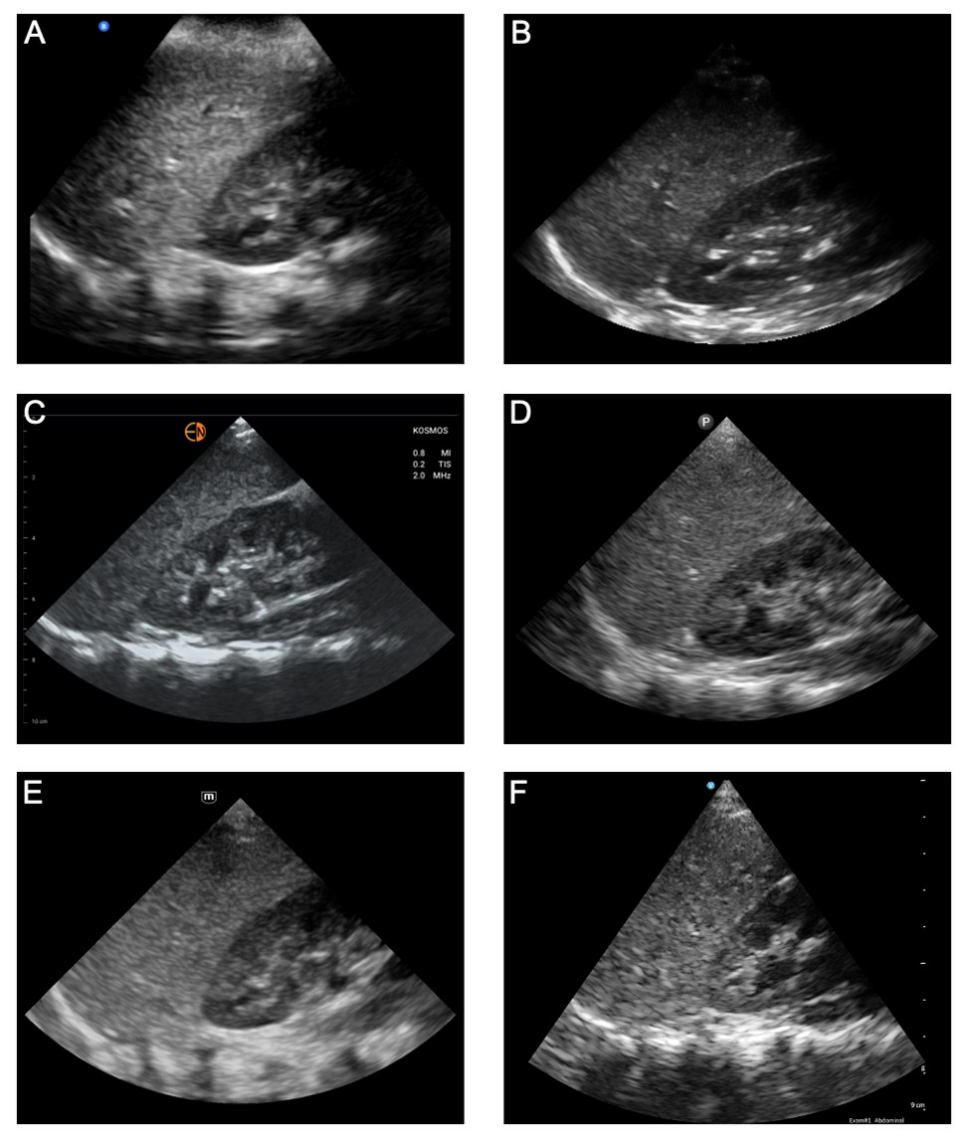
Abdominal Right Upper Quadrant Views acquired from the same pediatric model using six handheld ultrasound devices: (A) Butterfly iQ+™, (B) Clarius®, (C) Kosmos™, (D) Lumify™, (E) Mindray®, and (F) Vscan Air™.

**Figure 3B.**
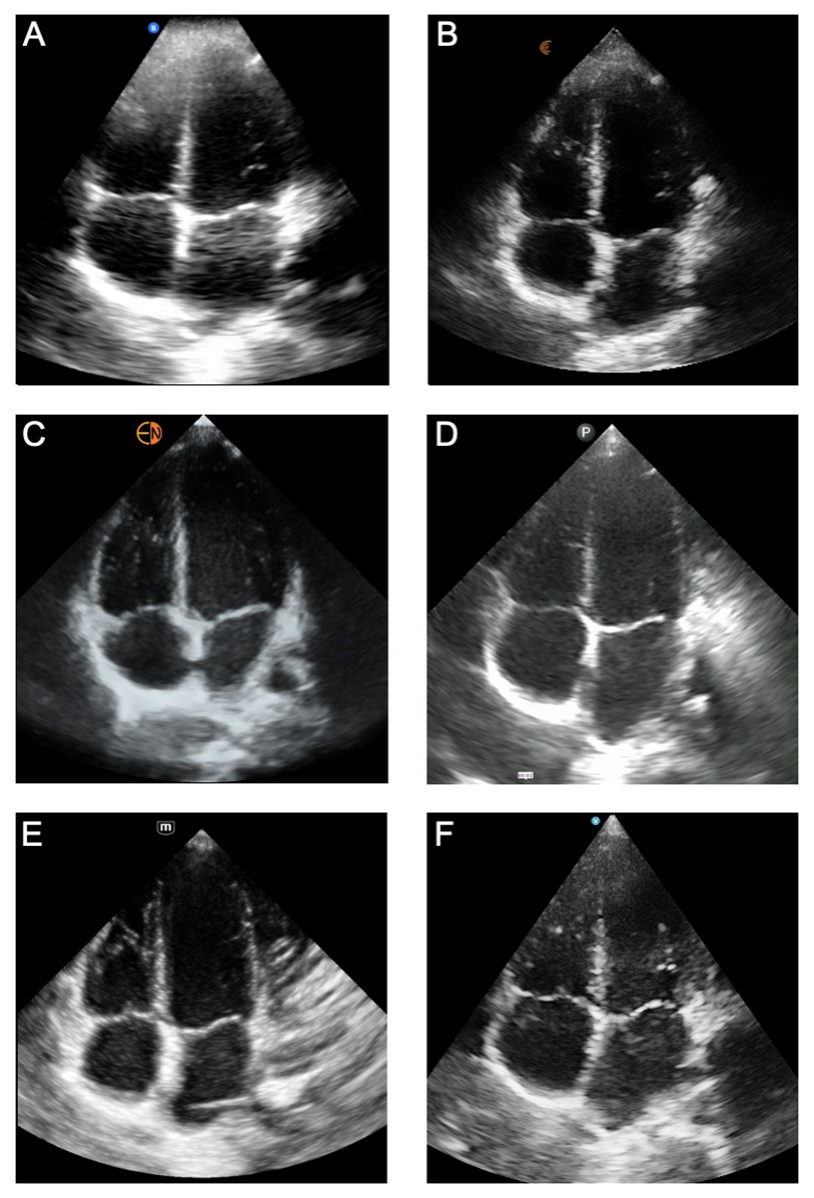
Cardiac Apical 4-Chamber Views acquired from the same pediatric model using six handheld ultrasound devices: (A) Butterfly iQ+™, (B) Clarius®, (C) Kosmos™, (D) Lumify™, (E) Mindray®, and (F) Vscan Air™.

**Figure 3C.**
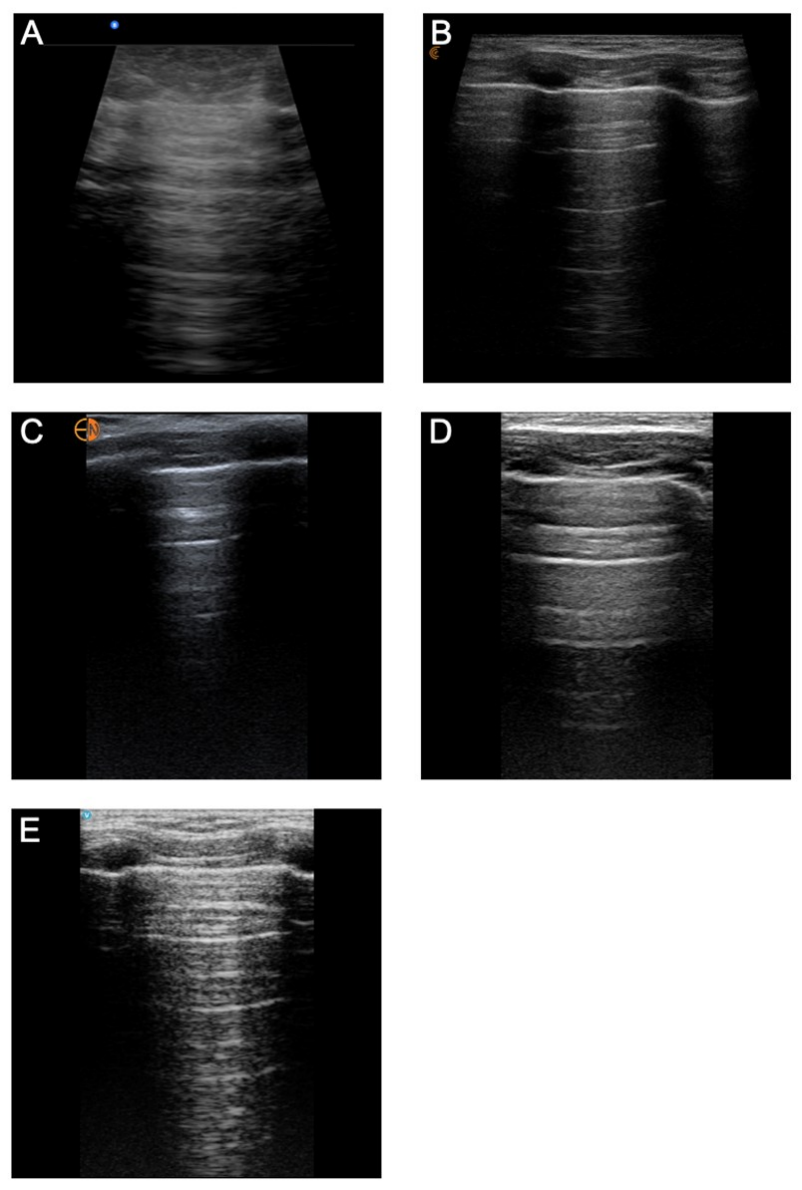
Superficial Neck Views acquired from the same pediatric model using six handheld ultrasound devices: (A) Butterfly iQ+™, (B) Clarius®, (C) Kosmos™, (D) Lumify™, (E) Vscan Air™.

**Figure 3D.**
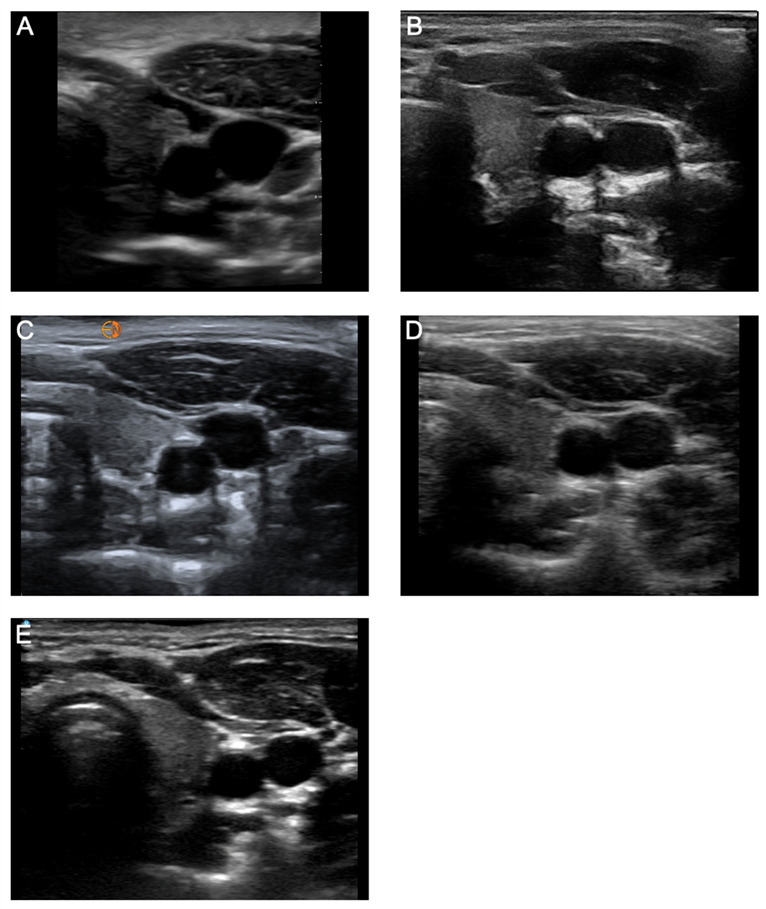
Lung Views of the same pediatric model using six handheld ultrasound devices: (A) Butterfly iQ+™, (B) Clarius®, (C) Kosmos™, (D) Lumify™, (E) Vscan Air™.

*Cardiac Apical 4-chamber View.* The apical 4-chamber view characteristics that were evaluated included endocardial definition, clarity of valve leaflets, clarity of the lateral tricuspid valve annulus, far-field resolution, and color flow Doppler of the left ventricular outflow tract and mitral valve (Figure 3; Appendix 5, Supplemental Table 2). For image quality, the handhelds with the highest image quality scores were the Kosmos™ (12.50), Vscan Air™ (12.38), Mindray® (11.75), Clarius® (10.38), and Lumify™ (10.38) — without a statistically significant difference between the devices. When ranked against each other, the devices finished in the same order. Butterfly iQ+™ was statistically inferior to other devices by total image quality score (6.00) and ranking score (11).

*Superficial Neck and Lung Views.* The superficial neck and lung view characteristics that were evaluated included the clarity of the carotid artery/internal jugular vein, color flow Doppler of carotid artery/internal jugular vein, difference in echogenicity of the thyroid gland, contrast of chest wall vs. pleural line, and clarity of lung sliding (Figure 3; Appendix 5, Supplemental Table 3). For image quality, the handhelds with the highest image quality scores were the Vscan Air™ (13.00), Clarius® (12.38), and Kosmos™ (12.13) — without a statistically significant difference between the devices. Lumify™ and the Butterfly iQ+™ were inferior for image quality of superficial structures. When ranked against each other, Vscan Air™ (39) ranked highest, followed by Kosmos™ (36) and Clarius® (33). Mindray® did not have a linear transducer and was excluded from the evaluation of superficial neck and lung views.

### Qualitative Data

Qualitative data gathered from experts revealed important themes (Table 5). After image quality, comments about transducer ergonomics (size, weight, and maneuverability) were common. Most importantly, the three highest ranking handhelds — Kosmos™, Lumify™, and Vscan Air™ — had more positive than negative comments, while Butterfly iQ+™, Clarius®, and Mindray® had more negative than positive comments, which was consistent with the quantitative data.

**Table 5.** Advantages and Disadvantages of Handhelds per Pediatric Point of Care Ultrasound Experts

	Advantages (% respondents)	Disadvantages (% respondents)
**Kosmos** ^**™**^	Good image quality (88%) Easy to use interface (38%) AI functions (38%)	Large probe size (50%) Multiple probes (13%) Poor image quality (13%) Difficult to use interface (13%) Cost (13%)
**Vscan Air** ^**™**^	Good image quality (75%) 2-in-1 probe (50%) Wireless connectivity (13%) Easy to use interface (13%) Cost (13%)	Connectivity problems (50%) Probe size/shape (50%) Difficult to use interface (13%) Dual probe (13%)
**Butterfly iQ+** ^**™**^	Easy to use interface (25%) 3-in-1 probe (25%) Cloud storage (25%) Cost (13%) Good image quality (13%)	Poor image quality (75%) Large probe size (63%) Membership fees (25%) Difficult to use interface (13%)
**Lumify** ^**™**^	Easy to use interface (63%) Good image quality (38%) Probe size (25%)	Multiple probes needed (50%) Probe size (38%) Poor image quality (13%) Limited spectral Doppler (no CWD) (13%)
**Mindray** ^®^	Good image quality (50%) Probe ergonomics (13%)	No linear probe (63%) Difficult to use interface (25%) Probe size/shape (25%) Poor image quality (13%)
**Clarius** ^**™**^	Good image quality (63%) Wireless (13%) Color Doppler (13%) Easy to use interface (13%)	Large probe (75%) Heat from probe (25%)

CWD, Continuous wave Doppler.

### Bias Evaluation

Potential bias due to prior experience with each handheld was assessed. Clarius® (1.13), Mindray® (1.13), and Kosmos™ (1.25) had mean experience scores <1.3, indicating minimal prior experience by experts. Vscan Air™ (1.5), Butterfly iQ+™ (1.75), and Lumify™ (1.75) had mean experience scores indicating some prior experience.

For most devices, no statistically significant association was found between prior handheld experience among experts and ratings of overall ease of use, image quality, and satisfaction (Appendix 6). In fact, no statistically significant association between experts' prior experience and their ratings of image quality was seen. For ease of use, the Vscan Air™ had a strong association with prior experience (correlation coefficient=0.93, p=0.001). Thus, experts with more Vscan Air™ experience tended to rate it as easier to use. For overall satisfaction, there was a positive association with prior experience for Clarius® (correlation coefficient=0.76, p=0.03). However, only one expert had some experience, suggesting minimal potential influence.

## Discussion

We conducted the first head-to-head comparative study by pediatric POCUS experts evaluating image quality, ease of use, and overall satisfaction of six commercially available handheld ultrasound devices in the United States. For image quality, ease of use, and overall satisfaction, Vscan Air™, Lumify™, and Kosmos™ were rated highest, and most pediatric POCUS experts selected Vscan Air™ as their preferred device to purchase. The five most desirable characteristics of handhelds were image quality, ease of use, total costs, transducer size, and availability of different transducer types.

Several past studies have assessed the utility of handhelds in pediatric populations for a broad range of applications, including rheumatic heart disease screening [3–7, 10, 23–30], pre-participation cardiac screening [31], evaluation of cardiac function [2, 8, 9, 32–34], and assessment of the bladder [11], muscles [12, 35], thyroid gland [13], internal jugular vein [14], venous malformations [18], and vocal cords [15]. Most of these studies compared handhelds to full-platform, cart-based ultrasound machines. They demonstrated similar, but not equivalent, diagnostic accuracy due to differences in operator experience, handheld characteristics, and diagnostic criteria (e.g., definite vs. borderline rheumatic heart disease).

Based on our literature review, our study is the first head-to-head comparison of handhelds with pediatric POCUS experts performing common POCUS exams of different body systems on a pediatric standardized patient. In adults, we identified four studies that directly compared handhelds head-to-head [19–22]. A study in 2020 compared three handhelds for gynecologic measurements and common pathologies, and concluded that Lumify™ was the best handheld overall in a resource-limited setting [36]. A recent study in 2024 evaluated five handhelds with three ophthalmologists acquiring views of facial arteries, ocular/periocular structures, and areas for filler injections in adults. It concluded that Clarius® L20 had the highest image quality for superficial facial structures [37]. Our group conducted head-to-head comparisons of handhelds with adult POCUS experts and standardized patients in 2022 and 2024. The overall satisfaction with image quality was rated highest with Vscan Air™ for the RUQ view, Mindray® for the cardiac apical 4-chamber view, and Lumify™ for the superficial views of the neck and lung. Vscan Air™ was the most preferred handheld for purchase in 2022 and 2024 [19, 22].

This comparative study of handhelds in pediatrics identified Vscan Air™, Kosmos™, and Lumify™ as the top three devices with the highest ratings for ease of use, image quality, and overall satisfaction. However, similar to studies comparing handhelds in adults, no single handheld was perceived as superior in image quality for all three views. In the present study, Butterfly iQ+™ was rated significantly lower in image quality compared to other handhelds for the RUQ, apical 4-chamber, and superficial views. Most importantly, our study revealed important advantages and disadvantages of each handheld in pediatric populations (Table 5). Several pediatric POCUS experts commented on their ability to comfortably hold and properly maneuver the handheld device (probe ergonomics).

Our study has important implications for future development and use of handhelds in pediatrics. Current handhelds have been developed for adult populations, and the transducer size, image resolution, and exam presets have not been optimized for pediatric and neonatal populations. Probe ergonomics was a major concern per qualitative feedback. Historically, ultrasound manufacturers have limited investments in developing devices specifically for pediatrics. However, handhelds give many pediatric clinicians access to ultrasound technology, and therefore play a critical role in pediatrics worldwide. For instance, several national rheumatic heart disease screening programs in Africa rely on the use of handhelds, which is evolving to become a standard of care in resource-limited settings [3–5, 7, 10, 23–30, 34]. At least one study commented on how the diagnostic accuracy of screening for rheumatic heart disease was dependent on the brand of handheld ultrasound used [7]. Furthermore, parents of children with cardiac transplants and other conditions can be trained to use handhelds to monitor their children at home. Incorporating artificial intelligence into these care models can improve remote monitoring of complex and chronically ill children [8, 9]. Finally, the development of handhelds configured for children will likely improve pediatric clinicians' access to ultrasound technology, allowing them to screen and triage children expeditiously. Eventually, position statements and guidelines on handhelds in children will emerge as they have in adults [38, 39].

This study has important strengths and limitations. First, we compared the six most common handhelds used in the United States per expert consensus, but not all available handhelds were compared, including the Vave Health and Exo handhelds. By having pediatric POCUS experts scan the same pediatric standardized patient using all six handhelds, we were able to minimize potential confounding from patient variables; however, additional standardized patients (neonates, infants, toddlers, and adolescents) with different body habitus would have ideally represented the broad range of pediatric patients. Additionally, experts evaluated the image quality of abdominal, cardiac, and superficial views using both high- and low-frequency transducers, similar to clinical practice. The eight pediatric POCUS experts were from six different institutions. They represented a diverse group of pediatric experts, including pediatric cardiology, critical care, emergency medicine, hospital medicine, and neonatology. Despite the diversity of specialties, we were limited to eight pediatric POCUS experts to evaluate the handhelds. With eight raters, we detected statistically significant differences of 1.5-2 points on a 5-point scale but would need 15-18 expert raters in future studies to detect significant differences of 1 point based on statistical modeling. In a bias analysis, no statistically significant association was found between prior handheld experience of the pediatric POCUS experts and their ratings of image quality (Appendix 6). Additionally, archiving handheld images on an institutional server in a picture archiving and communication system (PACS) or POCUS middleware is an important consideration when purchasing a handheld, especially for clinicians practicing in a hospital setting. Currently, most handhelds can export images in DICOM format to upload into a PACS, but the different POCUS workflows of each handheld — including image archiving, documentation, and billing — were not compared in this study.

## Conclusions

The three handhelds with highest ratings for ease of use, image quality, and overall satisfaction were Vscan Air™, Kosmos™, and Lumify™, per pediatric POCUS experts. When compared across different applications, no single handheld was clearly superior to the other handhelds, although a majority of pediatric POCUS experts selected the Vscan Air™ as their preferred device to purchase. The most desirable characteristics of handhelds were image quality, ease of use, total costs, transducer size, and availability of different transducer types. Several experts commented on probe ergonomics, reflecting concerns about use of handhelds created for adults in children with smaller body habitus. With similar ratings of image quality and ease of use, our study suggests that other qualitative characteristics, such as probe ergonomics, may be the distinguishing feature amongst handhelds that ultimately influence purchasing decisions. These findings may also serve as areas for improvement in future development of handhelds by ultrasound manufacturers.













## Device sources and industry involvement:

The Kosmos™ (EchoNous), Lumify™ (Phillips), Vscan Air™ SL (General Electric), TE Air™ (Mindray®), and Clarius Mobile Health™ ultrasound devices were temporarily loaned from their respective companies at the request of one of our authors (NJS). The Butterfly IQ+™ (Butterfly Network, Inc.) device was previously purchased by three of the study investigators (Abhilash Koratala, Tanping Wong, and Meghan K. Thomas) for personal use and temporarily loaned to conduct this study.

EchoNous, Philips, General Electric, Mindray®, Clarius Mobile Health™, and Butterfly Network, Inc. did not have any involvement in the development of this study, data review and analysis, writing of the manuscript, or any approval or decision-making in the submission of this manuscript. None of the authors are or were in any contractual agreement with EchoNous, Philips, General Electric, Mindray®, Clarius Mobile Health™, or Butterfly Network, Inc. regarding the study equipment. The authors did not receive any payment, support, or benefits in relation to this study from these companies. The Kosmos™ (EchoNous), Lumify™ (Phillips), Vscan Air™ (General Electric), TE Air™ (Mindray®), and Clarius Mobile Health™ ultrasound devices were temporarily loaned from their respective companies at the request of the senior author.
