# Supplementary material for: Comparison of Six Handheld Ultrasound Devices by Pediatric Point of Care Ultrasound (POCUS) Experts
Source: POCUS J. 2025 Apr 15;10(1):141–56. doi: 10.24908/pocusj.v10i01.18722 (PMC12057456; doi:10.24908/pocusj.v10i01.18722)
Supplement: Supplementary file 2 [file pocusj-10-01-18722-s002.pdf]

CARDIAC: APICAL 4-CHAMBER VIEW

IMAGE QUALITY RATING

RATER NAME: ________________________________

MODEL Station Number: ☐Handheld Apical 1 ☐Handheld Apical 2 ☐Handheld Apical 3

	**POOR = 0**<br>Inadequate Quality	**INTERPRETABLE = 1**<br>Minimally Adequate	**GOOD = 2**<br>Adequate Quality	**EXCELLENT = 3**<br>Superior Quality
Target Structures Visualized	Few	Some	All	All plus detail
Relative Image Quality	**Worse** than most handhelds	**Similar** to most handhelds	**Better** than most handhelds	**Similar to Cart-based** ultrasound machine
Ability to answer common clinical questions based on image	**NO!** No clinical questions could be answered with confidence	**Maybe** but some clinical questions could not be answered with confidence	**Yes,** most clinical questions could be answered with confidence	**YES!** All clnical questions could be answered with confidence
Could you make common clinical decisions based on this image?	"NO! I could **not make any clinical decisions** based on this image at all"	"Maybe – I could make **some clinical decisions** based on this image."	"Yes, I could make **most clinical decisions** with confidence based on this image."	"YES! I could make **all clinical decisions** with confidence based on this image."
Need to obtain additional ultrasound imaging with cart-based machine	**YES!** I definitely would need additional ultrasound imaging with a cart-based machine	**YES**, I most likely would need additional ultrasound imaging with a cart-based machine	**NO**, I most likely would NOT need additional ultrasound imaging with a cart-based machine	**NO!** I definitely would NOT need additional ultrasound imaging with a cart-based machine

Instructions:

1. Acquire an **apical 4-chamber view** on model designated as **"Handheld: Apical"** with the **phased-array probe** using a **cardiac exam** preset.
2. Use scale above (0-3) to **rate characteristics** of each handheld in the table below.
3. For "Overall Ranking," **rank the devices** from 1 ("Best") to 6 ("Worst") for this view.

	**Endocardial Definition** (0-3)	**Clarity of Valve Leaflets** (0-3)	**Clarity of Lateral TV Annulus** (0-3)	**Far-field Resolution** (0-3)	**Color Flow Doppler over LVOT or MV** (0-3)	**OVERALL RANKING (1=Best; 6=Worst)**
**Butterfly**						1 2 3 4 5 6
**Kosmos**						1 2 3 4 5 6
**Lumify**						1 2 3 4 5 6
**Vscan Air**						1 2 3 4 5 6
**Mindray**						1 2 3 4 5 6
**Clarius**						1 2 3 4 5 6
COMMENTS:						
