# Supplementary material for: Comparison of Six Handheld Ultrasound Devices by Pediatric Point of Care Ultrasound (POCUS) Experts
Source: POCUS J. 2025 Apr 15;10(1):141–56. doi: 10.24908/pocusj.v10i01.18722 (PMC12057456; doi:10.24908/pocusj.v10i01.18722)
Supplement: Supplementary file 5 [file pocusj-10-01-18722-s005.pdf]

Appendix 5. Image Quality Ratings of Specific Views

Supplemental Table 1. Image Quality Ratings based on the Abdomen Right Upper Quadrant View

View Characteristic Rated	Butterfly™	Clarius™	Kosmos™	Lumify™	Mindray™	Vscan Air™
Difference in echogenicity of renal cortex vs. liver[1]	2.00	2.25	2.63	2.38	2.63	2.50
Clarity of blood vessels in liver parenchyma	1.50	2.25	2.25	2.13	2.50	2.38
Distinguish medullary pyramids in renal cortex	1.25	2.38	2.00	1.50	2.25	1.88
Far-field resolution (spine, aorta)	1.88	2.13	2.50	2.38	2.25	2.25
Color flow Doppler of vessels in renal pelvis	1.75	2.25	2.00	2.00	2.38	2.38
**Total Image Quality Score[2]**	8.38	***11.25***	***11.38***	10.38	***12.00***	***11.38***
**Variability (s.d.)**	0.30	0.09	0.29	0.36	0.16	0.24
Summary of Rankings						
1st	0	1	3	1	1	2
2nd	1	2	1	0	3	1
3rd	0	0	2	2	1	2
4th	0	3	0	2	3	1
5th	0	2	2	3	0	1
6th	7	0	0	0	0	1
**Ranking Score[3]**	12	***29***	***35***	***26***	***34***	***31***

[1]For each characteristic the user could rate the device as Poor (Inadequate)=0, Interpretable (Minimally Adequate)=1, Good (Adequate)=2, or Excellent (Superior)=3. The value presented is the average of eight user ratings.
[2]Minimum possible total score is 0 and maximum possible total score is 15. Value presented is the total of the five means above it. The top score and any that are not significantly different from that score are designated in ***bold italic***.
[3]Minimum possible ranking score is 8 and maximum possible is 48. The top score and any that are not significantly different from that score are designated in ***bold italic***.

Supplemental Table 2. Image Quality Ratings based on the Cardiac Apical 4-chamber View

View Characteristic Rated	Butterfly™	Clarius™	Kosmos™	Lumify™	Mindray™	Vscan Air™
Endocardial definition[1]	1.00	1.88	2.38	1.88	2.13	2.38
Clarity of valve leaflets	1.50	2.25	2.50	2.38	2.63	2.63
Clarity of lateral tricuspid valve Annulus	1.25	2.38	2.50	2.25	2.63	2.63
Far-field resolution	1.13	2.00	2.63	2.00	2.25	2.63
Color flow Doppler over left ventricular outflow tract or mitral valve	1.13	1.88	2.50	1.88	2.13	2.13
**Total Image Quality Score[2]**	6.00	***10.38***	***12.50***	***10.38***	***11.75***	***12.38***
**Variability (s.d.)**	0.19	0.23	0.09	0.23	0.26	0.22
Summary of Rankings						
1st	0	0	5	1	1	1
2nd	0	2	0	0	2	4
3rd	0	1	1	2	2	2
4th	1	1	1	4	2	0
5th	1	4	1	0	1	1
6th	6	0	0	1	0	0
**Ranking Score[3]**	11	***25***	***39***	***27***	***32***	***36***

[1]For each characteristic the user could rate the device as Poor (Inadequate)=0, Interpretable (Minimally Adequate)=1, Good (Adequate)=2, or Excellent (Superior)=3. The value presented is the average of eight user ratings.

[2]Minimum possible total score is 0 and maximum possible total score is 15. Value presented is the total of the 5 means above it. The top score and any that are not significantly different from that score are designated in ***bold italic***.

[3]Minimum possible ranking score is 8 and maximum possible is 48. The top score and any that are not significantly different from that score are designated in ***bold italic***.

Supplemental Table 3. Image Quality Ratings based on Neck & Lung Sliding[1]

View Characteristic Rated	Butterfly™	Clarius™	Kosmos™	Lumify™	Vscan Air™
Clarity of carotid/internal jugular vein[2]	1.88	2.63	2.75	2.38	2.75
Color flow Doppler of carotid/internal jugular vein	2.38	2.25	2.75	2.25	2.88
Difference in echogenicity of thyroid, vs. vessels	1.88	2.63	2.38	1.75	2.50
Contrast of chest wall, vs. pleural line	2.13	2.38	2.25	2.25	2.38
Clarity of lung sliding ("shimmering" of pleural line)	2.25	2.50	2.00	2.38	2.50
**Total Image Quality Score[3]**	10.50	***12.38***	***12.13***	11.00	***13.00***
**Variability (s.d.)**	0.22	0.16	0.33	0.26	0.21
Summary of Rankings					
1st	1	2	1	0	4
2nd	0	1	5	1	1
3rd	0	1	0	5	2
4th	1	4	1	2	0
5th	6	0	1	0	1
**Ranking Score[4]**	21	33	36	31	39

[1]The Mindray device has no linear probe, so was unable to create a neck and lung sliding view and was not rated for this view.
[2]For each characteristic the user could rate the device as Poor (Inadequate)=0, Interpretable (Minimally Adequate)=1, Good (Adequate)=2, or Excellent (Superior)=3. The value presented is the average of eight user ratings.
[3]Minimum possible total score is 0 and maximum possible total score is 15. Value presented is the total of the 5 means above it. The top score and any that are not significantly different from that score are designated in ***bold italic***.
[4]Minimum possible ranking score is 16 and maximum possible is 48. The top score is not significantly different from the lowest score.
