# Supplementary material for: Comparison of Six Handheld Ultrasound Devices by Pediatric Point of Care Ultrasound (POCUS) Experts
Source: POCUS J. 2025 Apr 15;10(1):141–56. doi: 10.24908/pocusj.v10i01.18722 (PMC12057456; doi:10.24908/pocusj.v10i01.18722)
Supplement: Supplementary file 6 [file pocusj-10-01-18722-s006.pdf]

Appendix 6. Individual Expert's Experience with Devices Compared to Ratings for Overall Satisfaction, Image Quality, and Ease of Use

	Butterfly iQ+™				Clarius™				Kosmos™			
**Expert**	*Exp*	*Sat*	*Ease*	*Qual*	*Exp*	*Sat*	*Ease*	*Qual*	*Exp*	*Sat*	*Ease*	*Qual*
**1**	1	1	2.00	2.00	1	2	3.67	4.00	1	1	2.33	2.50
**2**	2	1	5.00	1.75	1	2	2.33	4.50	1	3	4.67	4.75
**3**	3	2	5.00	2.75	1	2	3.33	5.00	2	3	4.33	5.00
**4**	3	2	3.33	3.00	2	3	4.33	5.00	2	3	4.00	5.00
**5**	1	3	4.00	3.50	1	2	2.67	3.25	1	3	5.00	5.00
**6**	1	1	2.67	3.00	1	2	4.00	5.00	1	3	4.67	5.00
**7**	2	1	3.00	2.25	1	1	2.00	4.50	1	3	4.33	4.50
**8**	1	1	2.33	2.00	1	2	3.67	4.00	1	3	4.33	4.00
**r_S**		0.33	0.65	0.01		0.76	0.58	0.43		0.22	-0.39	0.54
**p-val**		0.43	0.08	0.98		0.03	0.13	0.63		0.60	0.34	0.17

	Lumify™				Mindray™				Vscan Air™			
**Expert**	*Exp*	*Sat*	*Ease*	*Qual*	*Exp*	*Sat*	*Ease*	*Qual*	*Exp*	*Sat*	*Ease*	*Qual*
**1**	1	1	3.00	4.00	1	2	3.67	4.00	1	3	3.33	4.00
**2**	2	3	5.00	4.50	1	2	3.00	2.75	2	3	5.00	4.75
**3**	2	2	4.00	3.75	2	2	5.00	4.75	2	3	5.00	5.00
**4**	1	3	4.33	5.00	1	2	4.00	4.25	2	3	5.00	4.00
**5**	1	3	4.00	3.75	1	2	3.33	3.00	1	2	2.33	2.25
**6**	2	3	5.00	4.75	1	1	3.00	3.00	1	2	3.67	5.00
**7**	2	3	5.00	5.00	1	2	4.33	4.50	2	3	5.00	5.00
**8**	3	3	4.33	3.25	1	1	2.67	3.00	1	3	4.67	4.00
**r_S**		0.26	0.52	-0.28		0.22	0.58	0.59		0.58	0.93	0.46
**p-val**		0.53	0.19	0.51		0.60	0.13	0.12		0.13	0.001	0.25

Exp*: Experience: 3=Extensive ("I use this device on a regular basis"), 2=Some ("I've used this device occasionally"), 1=None ("I've never used this device before").*

Sat*: Overall Satisfaction: 3=Satisfied ("I would use it in patient care"), 2=Neutral ("I might use it in patient care"), 1=Dissatisfied ("I would not use").*

Ease*: The average of 3 categories of Ease-of-Use, each ranked on a scale of 1 to 5, with 5 indicating the most satisfaction.*

Qual*: The average of 4 categories of Image Quality, each ranked on a scale of 1 to 5, with 5 indicating the most satisfaction.*

r_S *is the Spearman correlation coefficient, and* ***p-val*** *is the p-value of the test of association based on the Spearman correlation coefficient.*
